# Targeting copper death-related long non-coding RNAs: a novel strategy to overcome immunotherapy resistance in liver cancer

**DOI:** 10.3389/fimmu.2026.1743964

**Published:** 2026-03-02

**Authors:** Xiyang Sheng, Chen Mi, Gengyuan Shi, Longbo Wang, Yongzhao Li, Dongdong Wang, Wei Wang, Yongyue Du, Siyang Wang, Hanteng Yang

**Affiliations:** Department of General Surgery, Lanzhou University Second Hospital, Lanzhou, Gansu, China

**Keywords:** cuproptosis, hepatocellular carcinoma, immune evasion, immunogenic cell death (ICD), immunologicaltolerance, immunotherapy resistance, long non-coding RNA, tumor microenvironment

## Abstract

Hepatocellular carcinoma (HCC) is characterized by a profoundly immunosuppressive microenvironment that fosters active peripheral immune tolerance, thereby severely compromising the efficacy of immune checkpoint inhibitors (ICIs). The recent characterization of cuproptosis—a mitochondrial metabolism-dependent cell death—unveils a novel mechanistic link between metabolic stress and the disruption of this tolerance. This review elucidates the pivotal role of copper death-related long non-coding RNAs (lncRNAs) (CRLs) as epigenetic orchestrators that navigate the delicate balance between immune surveillance and tolerance. We systematically delineate the multifaceted mechanisms through which CRLs drive immune evasion and active immunological tolerance. Rather than a passive failure of the immune system to recognize the tumor, CRLs orchestrate a programmatic remodeling of the microenvironment. This involves (1) actively recruiting immunosuppressive populations, such as regulatory T cells (Tregs) and M2 macrophages, to establish immune exclusion; (2) synergistically upregulating co-inhibitory checkpoints (e.g., PD-L1) to induce effector T cell exhaustion; and (3) functioning as “molecular brakes” on cuproptosis-induced immunogenicity. Crucially, we highlight the functional heterogeneity of CRLs, identifying a distinct subset of tumor-suppressive lncRNAs (e.g., LINC02362) capable of promoting immunogenic cell death (ICD) to break established peripheral tolerance. Furthermore, we critically evaluate translational strategies, ranging from composite biomarkers to intelligent nanodelivery systems designed to precisely modulate the CRL axis. By shifting the paradigm from “drug resistance” to “tolerance modulation, “ this review provides a strategic roadmap for harnessing CRL-targeted interventions to restore immune homeostasis and sensitize HCC to immunotherapy.

## 1 Introduction

Hepatocellular carcinoma (HCC) poses a severe global public health challenge, with persistently high incidence and mortality rates (1). According to 2022 global cancer statistics, liver cancer ranks among the top three cancer-related causes of death in 46 countries and among the top five in 90 countries. The number of new cases worldwide is projected to continue increasing through 2050 (2). As HCC is the major subtype of primary liver cancer, most patients are diagnosed at an advanced stage, facing limited treatment options and poor overall prognosis (3). Current therapeutic approaches include surgical resection, liver transplantation, local ablation, interventional therapy, and molecular targeted drug therapy (4). Although tyrosine kinase inhibitors (such as sorafenib and lenvatinib) have provided some survival benefit for advanced patients, their overall efficacy remains limited (5).In recent years, immunotherapy, represented by immune checkpoint inhibitors, has revolutionized the treatment landscape for advanced HCC, significantly improving clinical outcomes for some patients (6). Nevertheless, objective response rates remain low, and both primary and acquired resistance are prevalent, constituting key bottlenecks limiting further clinical efficacy improvements (7). Therefore, deepening the understanding of immune resistance mechanisms in HCC and developing novel reversal strategies represent core scientific challenges that require resolution in this field.

Immune checkpoint inhibitors (ICIs), primarily targeting the programmed death protein-1 (PD-1) and its ligand (PD-L1), have become cornerstones of systemic therapy for advanced HCC, demonstrating notable efficacy, especially in combination with anti-angiogenic agents (8, 9). The IMbrave150 trial established that the atezolizumab (anti-PD-L1) plus bevacizumab (anti-VEGF) combination significantly prolonged overall survival compared to sorafenib, heralding a new era of combination immunotherapy for HCC (10, 11). However, the overall response rate to immunotherapy remains suboptimal, with approximately 30% of patients exhibiting primary resistance. Moreover, most initial responders develop acquired resistance during subsequent treatment, ultimately leading to disease progression (6, 12). Current research indicates that factors such as the infiltration of immunosuppressive cells (e.g., M2 macrophages, CD10+ALPL+ neutrophils) in the tumor immune microenvironment, the functional exhaustion of effector T cells, and non-coding RNAs derived from exosomes collectively form a complex immunosuppressive network that significantly diminishes the actual efficacy of immunotherapy (13). Therefore, systematically deciphering the mechanisms of HCC immune resistance and developing effective reversal strategies have become focal scientific challenges in this field.

Cuproptosis is a recently identified, copper ion-dependent modality of programmed cell death, distinct in its molecular mechanisms from other forms such as apoptosis, necroptosis, autophagy, and ferroptosis (14) (**Table 1**). This process relies on abnormal intracellular copper accumulation and induces toxic protein oligomerization and aggregation by specifically binding to acyl-modified metabolic enzymes in the tricarboxylic acid (TCA) cycle, ultimately triggering lethal protein stress responses (15). Research indicates that copper homeostasis plays a dual role in tumorigenesis and progression, making targeted induction of Cuproptosis a highly promising novel antitumor strategy (16). Crucially, copper metabolism is intricately linked to regulating the tumor immune microenvironment—copper ions directly modulate immune cell functional states and immune checkpoint molecule expression (17, 18). This suggests that targeting Cuproptosis pathways may offer a novel intervention strategy to reverse tumor immune suppression and enhance immunotherapy sensitivity.

**Table 1:** Comparison of different types of cell death.

Types of cell death	Definition	Characteristics	Key markers
Cuproptosis (25, 26)	Cuproptosis is a novel form of cell death regulation triggered by intracellular copper accumulation	Mitochondrial atrophy, mitochondrial membrane rupture	FDX1, DLAT, LIAS, HSP70
Ferroptosis (27, 28)	Ferroptosis is a form of cell death induced by iron accumulation and oxidative stress	Mitochondrial atrophy, mitochondrial membrane rupture, reduced mitochondrial cristae	Fe, GSH, MDA, GPX4, ROS
Apoptosis (29)	Apoptosis is a programmed cell death process mediated by apoptotic bodies and caspases, aimed at maintaining cellular homeostasis	cell shrinkage, chromatin condensation, membrane integrity preserved, apoptotic body formation	P53, BCL-2
Pyroptosis (30)	Pyroptosis is a form of programmed cell death triggered by inflammasomes	Cell swelling, cell membrane rupture, DNA condensation and fragmentation, release of pro-inflammatory cytokines	IL-18, Caspase 1, Gasdermin D, IL-1
Necroptosis (31, 32)	A programmed form of necrosis, mediated by RIPK1, RIPK3, and MLKL	Cell swelling, plasma membrane rupture, leakage of intracellular contents, strong inflammatory response	p-RIPK1, p-RIPK3, p-MLKL, MLKL oligomers
Autophagy (33, 34)	A catabolic process involving lysosomal degradation of cytoplasmic components; excessive autophagy can lead to cell death	Extensive cytoplasmic vacuolization (formation of autophagosomes), organelle degradation, lack of chromatin condensation	LC3-II (conversion from LC3-I), Beclin-1, p62 (degradation)

Long noncoding RNAs (lncRNAs) are defined as RNA transcripts longer than 200 nucleotides that typically lack protein-coding potential (19). They play pivotal gene regulatory roles at epigenetic, transcriptional, and post-transcriptional levels via diverse mechanisms, such as modulating chromatin states, influencing transcription, regulating RNA stability, and functioning as competitive endogenous RNAs (ceRNAs). These molecules are deeply involved in core biological processes such as cell proliferation, metabolic reprogramming, and immune responses (20, 21). Within the tumor microenvironment, lncRNAs not only regulate the metabolic characteristics of tumor cells themselves but also remotely modulate macrophage polarization, T cell function, and immune checkpoint molecule expression through vesicular transport systems such as exosomes, thereby profoundly reshaping the tumor immune landscape (22). Growing evidence indicates that lncRNAs play a pivotal role in the initiation, progression, and immune evasion of HCC, suggesting their potential as novel targets for reversing immune therapy resistance in HCC.

Therefore, copper death-related lncRNAs (CRLs) emerge as pivotal molecular nodes linking metabolic cell death with tumor immune regulation, offering a novel conceptual framework and a compelling research direction for dissecting the mechanisms of immunological tolerance and resistance in HCC (23). Research indicates that CRLs not only modulate HCC cells’ sensitivity to copper-induced death but also actively orchestrate immune evasion by fostering a tolerogenic microenvironment. This dual role positions them as biomolecules with both prognostic predictive value and therapeutic target potential (24). This comprehensive review sets out to systematically decipher how CRLs orchestrate the mechanisms of immunological tolerance and resistance in HCC. We first delineate the core mechanisms of cuproptosis and its interplay with antitumor immunity. We then elaborate on the specific pathways through which CRLs facilitate immune evasion and enforce tolerance, and finally, we critically evaluate the translational prospects of CRLs as biomarkers and therapeutic targets. By synthesizing extant knowledge and highlighting emerging trajectories, this review aims to establish a robust foundation for developing novel CRL-directed precision therapies, with the ultimate goal of restoring immune homeostasis and augmenting immunotherapy outcomes in HCC. In this review, “tumor-induced tolerance” is operationally reflected by (i) enrichment of suppressive populations (Tregs/M2-TAMs/TANs), (ii) impaired antigen presentation and DC maturation, and (iii) sustained effector dysfunction with an exhaustion/checkpoint program (e.g., PD-1/LAG-3/TIM-3) rather than a simple lack of immune recognition.

## 2 Core mechanisms of cuproptosis and its interplay with antitumor immunity

Immunotherapy resistance in hepatocellular carcinoma constitutes a complex, multifactorial problem, involving tumor cell heterogeneity, an immunosuppressive microenvironment, and metabolic reprogramming, among other factors (7). The recent discovery of cuproptosis, a novel form of programmed cell death, offers a new perspective on this challenge (35). Distinct from established cell death pathways, cuproptosis is uniquely dependent on mitochondrial respiration and the accumulation of toxic copper ions. Its mechanism is intimately linked to the metabolic characteristics of tumor cells and unveils a broad and complex immunoregulatory potential (14).This section examines the core molecular mechanisms of cuproptosis and systematically explores its interplay with the tumor immune microenvironment (TME), thereby establishing a theoretical foundation for understanding its role as a novel strategy to overcome immunotherapy resistance in hepatocellular carcinoma.

### 2.1 Molecular mechanisms of cuproptosis: from copper ion accumulation to proteotoxic stress

**Figure 1:**
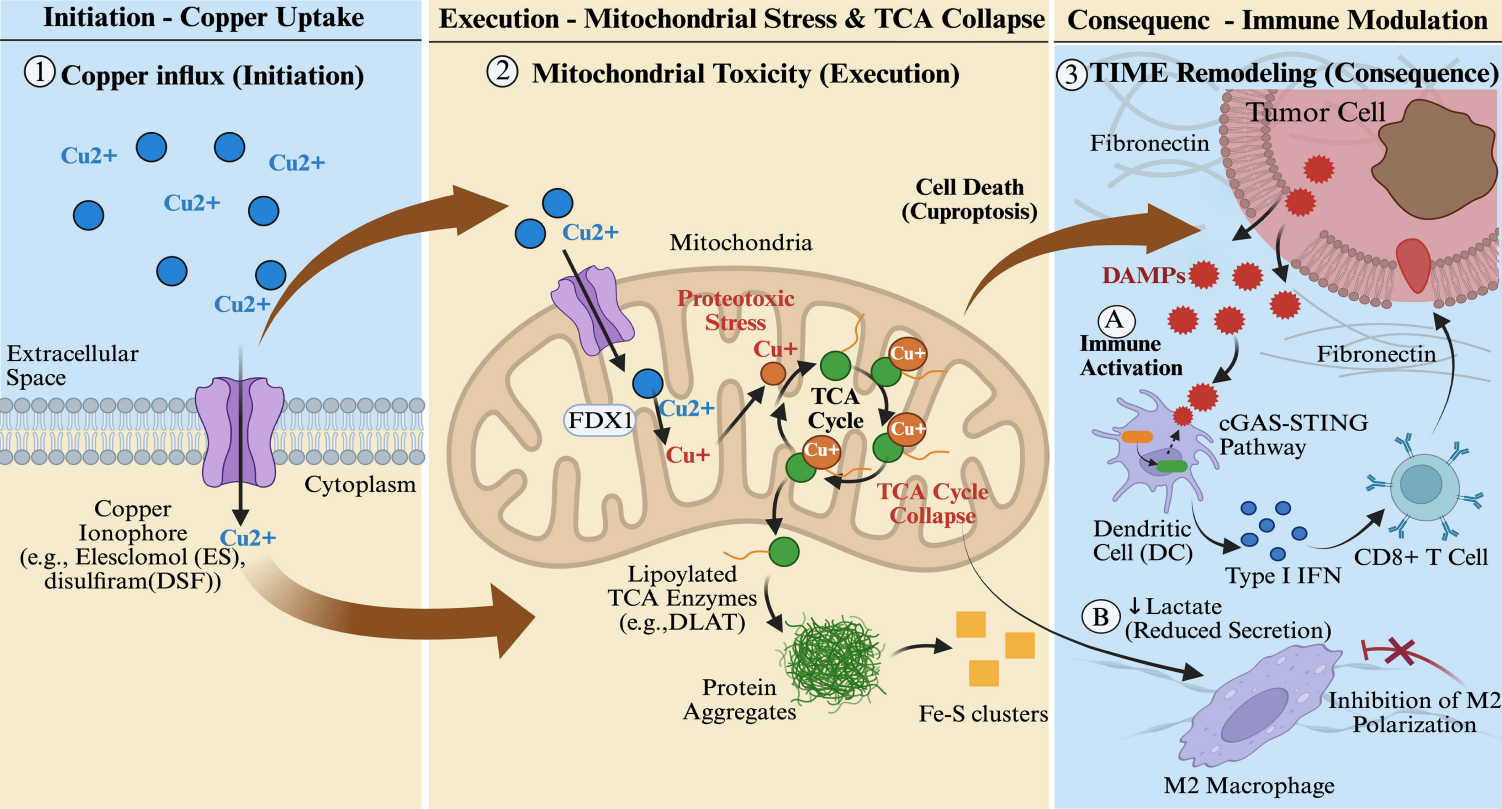
Sequential mechanism of copper-induced cell death and its remodeling of the tumor immune microenvironment.

Cuproptosis is executed through a precise, multi-step biological process. It is initiated by the abnormal accumulation of copper ions within mitochondria, which triggers a cascade of reactions culminating in lethal proteotoxic stress (15) (**Figure 1**).

#### 2.1.1 FDX1-mediated copper reduction and metabolic enzyme targeting

Cuproptosis begins with the disruption of intracellular copper homeostasis (36). Dietary Cu²^+^ enters cells primarily through the high-affinity copper transporter SLC31A1 (CTR1) (37). Upregulation of SLC31A1 in hepatocellular carcinoma has been linked to oncogenic signaling pathways, suggesting a potential vulnerability of HCC cells to cuproptosis (38). Copper ionophores (e.g., Elesclomol and disulfiram) facilitate efficient copper delivery by forming complexes with copper ions and promoting their specific mitochondrial accumulation (39).Within the mitochondrial matrix, ferredoxin-1 (FDX1) serves as a central player. FDX1 acts not only as the principal reductase converting Cu²^+^ to the more toxic Cu^+^ but also as a key upstream regulator of the entire cuproptosis pathway (40). Genome-wide CRISPR screens confirm that FDX1 loss confers robust resistance to various copper ionophores, highlighting its essential role (41).

#### 2.1.2 Oligomerization of acylated DLAT

The reduced Cu^+^ does not indiscriminately damage cellular components but displays high specificity, primarily targeting key lipoylated enzymes within the tricarboxylic acid (TCA) cycle (42). This precision arises from the exceptionally high affinity of Cu^+^ for protein lipoyl groups, with dissociation constants on the order of 10^-17M (41). Among these, the E2 component of the pyruvate dehydrogenase complex—dihydropyrrolidine thioamide acyltransferase (DLAT) (35)—has been identified as a core target for Cu^+^ action (43). Notably, FDX1 plays a dual central role in this process: it serves not only as the key reductase catalyzing the reduction of Cu²^+^ to highly reactive Cu^+^, but also as a positive regulator of protein lipoylation (15). Studies indicate that FDX1 promotes the lipoylation modification of mitochondrial proteins such as DLAT by directly interacting with lipoic acid synthase (LIAS) (39). Consistently, FDX1 knockout ablates cellular protein lipoylation and leads to significant accumulation of TCA cycle intermediates, including pyruvate and α-ketoglutarate (25).These findings position FDX1 as the central node connecting copper toxicity to mitochondrial metabolic enzyme dysfunction.

#### 2.1.3 Protein toxicity stress, iron-sulfur cluster loss, and cell death execution

The binding of Cu^+^ to lipoylated DLAT induces abnormal, disulfide-dependent oligomerization and insoluble aggregation of the protein, rather than simply inhibiting its activity. These aggregates appear as distinct protein foci under electron microscopy, marking the physical collapse of the central TCA cycle (15).This process coincides with the destabilization and substantial loss of mitochondrial iron-sulfur (Fe-S) cluster proteins. As essential cofactors for enzymes involved in oxidative phosphorylation and DNA repair, the loss of Fe-S clusters severely exacerbates mitochondrial dysfunction (38). Ultimately, the coalescence of lipoylated protein aggregates and Fe-S cluster degradation triggers intense mitochondrial proteotoxic stress. This is marked by the induction of heat shock proteins (e.g., HSP70), but the cell fails to recover and undergoes death (42).A key feature of cuproptosis is its strong dependence on active mitochondrial respiration. Consequently, cells reliant on glycolysis or located in hypoxic niches exhibit significant resistance, providing a rationale for selectively targeting tumor cells with robust mitochondrial respiration and TCA cycle turnover.

### 2.2 Interactions between cuproptosis and the tumor immune microenvironment

The antitumor effects of cuproptosis extend beyond direct cancer cell cytotoxicity to include its function as a potent cellular stress signal. This signal profoundly remodels the tumor immune microenvironment (TME) and engages in multifaceted, complex crosstalk with the host antitumor immune response (44).

#### 2.2.1 Immunogenic cell death and antitumor immune activation

Inducing cuproptosis may reverse tumor-induced immunosuppression, potentially converting immunologically “cold” tumors into “hot” tumors with enhanced immune cell infiltration, thereby improving responses to immunotherapy (42).Preclinical studies demonstrate that cuproptosis induced by Elesclomol-Cu or Disulfiram-Cu effectively activates the cGAS-STING innate immune pathway in dendritic cells (DCs). This activation promotes DC maturation and antigen presentation capacity and stimulates the secretion of proinflammatory factors like type I interferons, collectively initiating robust tumor antigen-specific CD8^+^ T cell responses. This process lays a critical immunological foundation for the efficacy of immune checkpoint inhibitors (38).

#### 2.2.2 Metabolic reprogramming reverses immunosuppression

Cuproptosis directly disrupts tumor cell metabolic reprogramming by targeting the TCA cycle, thereby altering the TME’s metabolite composition (26).For example, TCA cycle inhibition reduces tumor cell lactate production. As a key immunosuppressive metabolite in the TME, lactate directly impairs the function and proliferation of cytotoxic T cells and NK cells while promoting the activation and recruitment of immunosuppressive cells, such as M2 macrophages and regulatory T cells (Tregs) (15). Therefore, by reducing lactate levels in the TME, cuproptosis-induced metabolic reprogramming can indirectly alleviate the suppression of effector immune cells, helping to reverse the overall immunosuppressive state.

#### 2.2.3 Bidirectional regulation of immune checkpoints by copper homeostasis

A close but complex relationship exists between copper metabolism and immune checkpoint molecule expression. Substantial evidence indicates that elevated intracellular copper levels upregulate PD-L1 expression, influencing both its transcription and protein stability, potentially through copper-mediated activation of STAT3 and EGFR signaling pathways (45). This suggests that cuproptosis induction as a monotherapy could paradoxically increase PD-L1 expression in surviving tumor cells, potentially enhancing their immune evasion and fostering adaptive resistance (45).

However, this elevated PD-L1 expression simultaneously provides an increased density of therapeutic targets, potentially rendering surviving tumor cells more susceptible to anti-PD-L1 monoclonal antibodies (mAbs). This dynamic interplay underscores the synergistic potential of combining cuproptosis induction with immune checkpoint blockade to effectively eliminate drug-tolerant persister cells (45, 46). Conversely, this interplay supports combination therapy: using copper chelators (e.g., tetrasulfomolybdate) or modulating cuproptosis can promote PD-L1 ubiquitination and degradation, thereby enhancing the infiltration of CD8^+^ T cells and NK cells into tumors (45). Therefore, precise modulation of cuproptosis and its associated pathways represents a promising strategy to concurrently eliminate tumor cells and reduce immune checkpoint barriers, achieving synergistic antitumor effects.

The mechanism is illustrated in three sequential phases (1): Initiation - Copper Uptake (Left): Copper ionophores, represented by purple transmembrane channels (e.g., elesclomol (ES) and disulfiram (DSF)), facilitate the influx of extracellular Cu²^+^ (blue spheres) into the cytoplasm and subsequently into the mitochondria (2). Execution - Mitochondrial Toxicity (Center): Within the mitochondrion, FDX1 reduces Cu²^+^ to Cu^+^ (orange spheres). The highly reactive Cu^+^ selectively binds to lipoylated TCA cycle enzymes (depicted as green spheres with lipid tails, e.g., DLAT). This binding triggers the formation of insoluble protein aggregates (shown as green tangled structures) and the loss of Fe-S clusters (yellow squares), leading to proteotoxic stress and the physical collapse of the TCA cycle (3). Consequence - Immune Modulation (Right): The rupture of the dying tumor cell releases DAMPs (red spiky shapes), which are engulfed by Dendritic Cells (DCs) to activate the cGAS-STING pathway, promoting Type I IFN secretion and CD8^+^ T cell infiltration (Path A). Concurrently, the collapse of the TCA cycle results in reduced lactate secretion (↓ Lactate), thereby attenuating lactate-driven M2 polarization/inhibiting M2 polarization (Path B).

## 3 LncRNAs: bridging cuproptosis and immunotherapy resistance

### 3.1 Identification, molecular heterogeneity, and pathophysiological significance of cuproptosis-related lncrnas in hepatocellular carcinoma

The identification of cuproptosis-related lncRNAs (CRLs) represents a systematic endeavor that integrates bioinformatics mining with experimental validation. Currently, the recognition of CRLs is primarily based on two fundamental principles: expression correlation and functional association (47). In hepatocellular carcinoma (HCC) research, the prevailing strategy utilizes public databases (e.g., TCGA-LIHC) (48)to preliminarily screen candidate molecules by calculating expression correlation coefficients between genome-wide lncRNAs and established core cuproptosis regulators, such as FDX1, DLAT, and LIAS. Subsequently, through the integrative application of differential expression analysis (HCC tissues vs. adjacent non-tumor tissues) (49), survival prognostic modeling (50), and multi-omics network analysis (e.g., co-expression and ceRNA network construction) (51), researchers further pinpoint lncRNAs that exhibit aberrant expression, possess independent prognostic value, and are deeply implicated in core biological pathways within HCC. Finally, the biological functions and clinical significance of these candidates are validated through the construction of multi-gene prognostic signatures (e.g., CRDELSig), complemented by *in vitro* and *in vivo* gain- and loss-of-function experiments (52).

Accumulating evidence indicates that CRLs exhibit significant functional heterogeneity within the context of HCC. Their biological effects are not monolithically oncogenic or tumor-suppressive; rather, they display a high degree of context-dependence, governed by their expression abundance, interaction networks, and downstream effector pathways (**Table 2**). This heterogeneity directly dictates their regulatory efficacy in shaping the response to immunotherapy.

**Table 2:** Expression profiles, prognostic associations, and potential functional mechanisms of key copper death-related lncRNAs (CRLs) in cancer.

LncRNA name	Expression status	Association of high expression with prognosis	Primary functional mechanism
MKLN1-AS (43, 53, 54)	Significantly Upregulated	Poor	Acts as a ceRNA for miR-654-3p, upregulating HDGF expression to drive HCC malignant progression; serves as a key component of CRDELSig; its knockdown upregulates FDX1, participating in copper-mediated apoptosis
AC026412.3 (55)	Upregulated	Poor	Key member of CRL prognostic signature; predicts immunotherapy response
TMCC1-AS1 (56)	Upregulated	Poor	Promotes HCC cell proliferation, migration, invasion, and EMT; common component in multiple prognostic models
AL133243.2 (53)	Upregulated	Adverse	High-risk group characteristic lncRNA; positively correlated with immune checkpoint expression; inhibits NK cell activation
KDM4A-AS1 (52, 54)	Significantly Upregulated	Poor	Promotes tumor growth and metastasis via miR-411-5p/KPNA2/AKT axis; as a key component of CRDELSig, its knockdown upregulates FDX1 and participates in Cuproptosis processes
LINC02362 (51)	Downregulated	Good	Sponges miR-18a-5p to upregulate FDX1; *promotes* cuproptosis and enhances oxaliplatin sensitivity.

#### 3.1.1 Oncogenic CRLs: drivers of tumor progression and immunological tolerance

These oncogenic CRLs are characteristically upregulated in HCC and are intimately linked to poor patient prognosis, the establishment of an immunosuppressive microenvironment, and resistance to therapy. Their primary oncogenic mechanisms include:

Suppression of Cuproptosis Sensitivity: Certain CRLs function as negative regulators of key cuproptosis genes via transcriptional or post-transcriptional mechanisms, thereby dampening the susceptibility of tumor cells to copper-induced cell death. For instance, MKLN1-AS and KDM4A-AS1—key components of the prognostic model CRDELSig—are significantly upregulated in HCC. Knockdown of these molecules has been shown to restore FDX1 expression and inhibit cell proliferation, suggesting their roles as negative regulators of the cuproptosis machinery (52) (54).

Activation of Oncogenic Signaling via ceRNA Mechanisms: A subset of CRLs functions as competitive endogenous RNAs (ceRNAs), specifically sequestering miRNAs to relieve the repression of downstream oncogenes. Specifically, MKLN1-AS competitively binds to miR-654-3p, thereby upregulating the expression of hepatoma-derived growth factor (HDGF) to drive the malignant evolution of HCC (54).

Remodeling the Immune Microenvironment Landscape: Multiple studies have confirmed that high-risk scores based on CRL signatures are positively correlated with the infiltration abundance of immunosuppressive populations, such as M2 macrophages and regulatory T cells (Tregs), within the tumor microenvironment. Concurrently, these scores are associated with the downregulation of CD8^+^ T cell and natural killer (NK) cell activity (53, 57). In particular, the overexpression of AL133243.2 exhibits a significant negative correlation with functional markers of NK cells, indicating its involvement in the functional suppression of effector immune cells (53).

#### 3.1.2 Putative tumor-suppressive CRLs

In contrast to the aforementioned resistance-driving oncogenic molecules, a subset of CRLs is significantly downregulated in HCC, and their elevated expression correlates positively with favorable prognosis and therapeutic sensitivity. This observation highlights the marked heterogeneity of CRLs in modulating the direction of cuproptosis.

LINC02362 exemplifies this category of molecules. Research confirms that LINC02362 is significantly downregulated in HCC tissues, with its low expression linked to adverse clinicopathological characteristics. Mechanistically, LINC02362 functions as a competitive endogenous RNA (ceRNA) for miR-18a-5p, thereby relieving the post-transcriptional repression of the core cuproptosis gene FDX1. The overexpression of LINC02362 restores FDX1 levels, thereby sensitizing tumor cells to cuproptosis induced by copper ionophores (e.g., Elesclomol) and synergistically enhancing the antitumor efficacy of oxaliplatin (51). Crucially, this discovery demonstrates that specific CRLs can function as “accelerators” rather than “brakes” of the cuproptosis program, providing direct evidence for developing CRL-based sensitization strategies.

### 3.2 Specific mechanisms and functional heterogeneity of CRLs driving immunotherapy resistance

**Figure 2:**
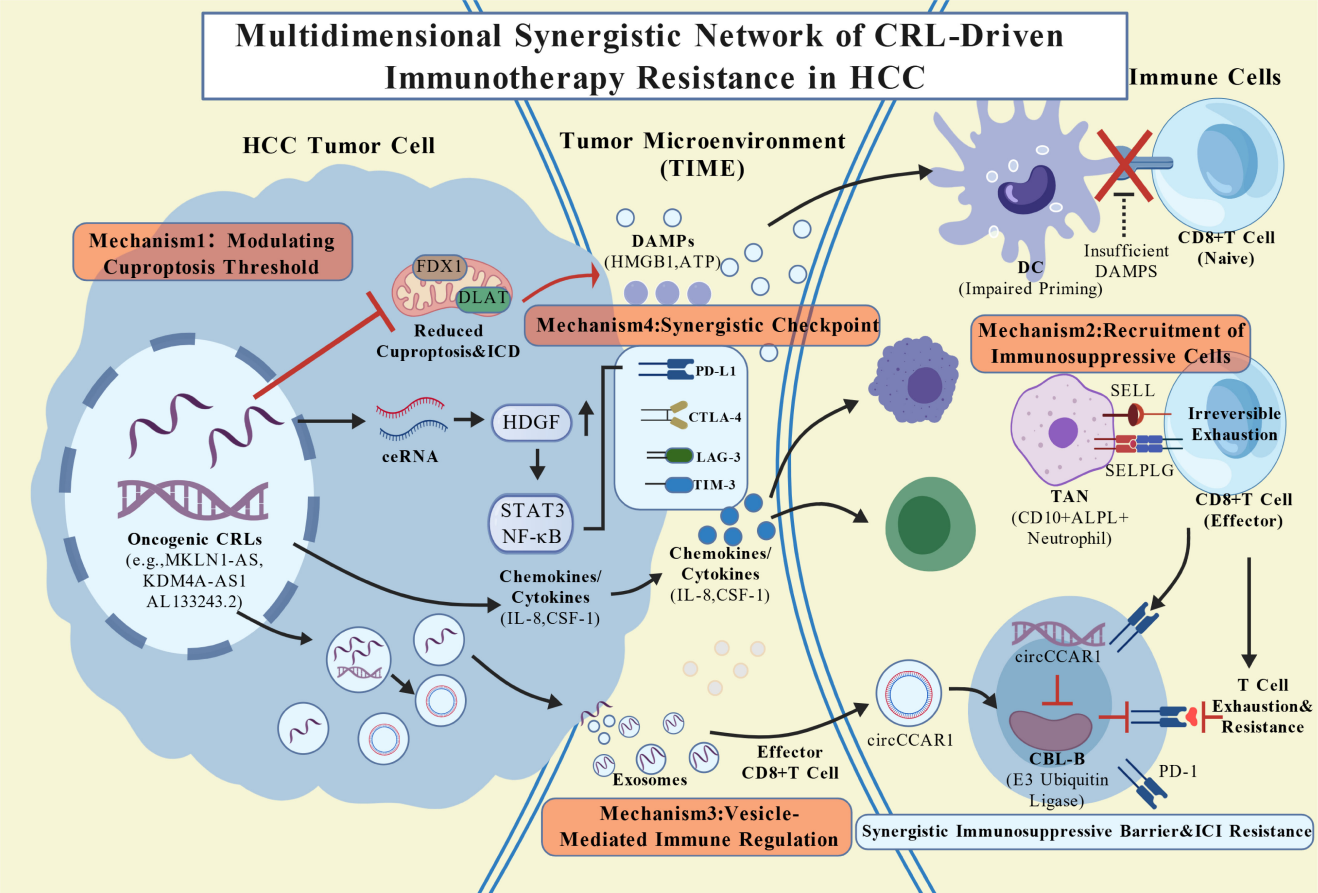
Schematic diagram of the multidimensional mechanism by which copper death-associated lncRNAs drive immune resistance in hepatocellular carcinoma. Mechanism 1 (Top Left/Right): CRLs downregulate cuproptosis machinery (FDX1/DLAT), reducing ICD and DAMPs release, impairing DC-mediated T cell priming. Mechanism 2 (Middle): CRLs drive cytokine networks to recruit suppressive cells (TANs, M2, Tregs). TANs directly induce T cell exhaustion via SELL-SELPLG interaction. Mechanism 3 (Bottom Right): Exosomal CRLs (e.g., circCCAR1) transfer to T cells, inhibiting CBL-B to stabilize PD-1 receptors, driving exhaustion. Mechanism 4 (Integrated Surface): CRLs synergistically upregulate inhibitory checkpoints (e.g., PD-L1 on tumor, PD-1 stability on T cells), creating a multidimensional immune barrier that drives exhaustion while simultaneously offering an increased density of therapeutic targets for combinatorial immunotherapy. While this schematic primarily illustrates the dominant resistance mechanisms driven by oncogenic CRLs, the potential for tumor-suppressive CRLs (e.g., LINC02362) to promote antitumor immunity via FDX1 upregulation is discussed in detail in Section 3.2.2.

Cuproptosis-related long non-coding RNAs (CRLs) reshape the tumor immune microenvironment (TIME) by constructing a multidimensional, synergistic, and highly complex regulatory network, thereby driving resistance to immune checkpoint inhibitors (ICIs) in hepatocellular carcinoma (HCC) (53) (**Figure 2**). These mechanisms are intertwined, collectively establishing a niche that suppresses antitumor immune responses. Notably, existing evidence underscores significant functional heterogeneity among different CRLs. Their ultimate impact on immunotherapy response depends on their regulatory direction regarding the cuproptosis pathway (promotion vs. inhibition), the specific cell types involved, and local microenvironmental signals (51, 54).

#### 3.2.1 Core resistance mechanisms: a multidimensional synergistic network

##### 3.2.1.1 Mechanism 1: modulating the cuproptosis threshold and immunogenic determinants

CRLs directly influence tumor cell death sensitivity and subsequent immunogenicity by precisely regulating the expression of core cuproptosis execution molecules. Oncogenic or resistance-associated CRLs (e.g., MKLN1-AS, KDM4A-AS1) frequently serve as negative regulators of the cuproptosis pathway. Through mechanisms such as transcriptional repression, epigenetic silencing, or functioning as competitive endogenous RNAs (ceRNAs), these lncRNAs downregulate the expression of key proteins like FDX1 and DLAT (54, 57). This elevation of the “death threshold” not only promotes cell survival but, crucially, attenuates immunogenic cell death (ICD) triggered by cuproptosis. Consequently, the reduction in ICD diminishes the release of damage-associated molecular patterns (DAMPs, such as HMGB1 and ATP) and subsequent antigen presentation by dendritic cells (DCs), leading to insufficient priming of CD8^+^ T cells. Clinical correlation analyses indicate that high-risk scores based on CRL signatures are significantly associated with reduced density of CD8^+^ T cell infiltration in HCC tissues (52, 54, 57).

##### 3.2.1.2 Mechanism 2: recruitment and reprogramming of immunosuppressive cell populations

CRLs actively shape an immunosuppressive cellular ecology by regulating the expression networks of chemokines, cytokines, and their receptors. For instance, the high expression of specific CRLs (e.g., AL133243.2) is correlated with the activation of signaling pathways that enrich tumor-associated neutrophils (TANs), M2 macrophages, and regulatory T cells (Tregs) (53, 58). Recent findings indicate that a distinct CD10^+^ALPL^+^ neutrophil subset is enriched in HCC patients resistant to ICIs. These neutrophils interact with T cells via the SELL (L-selectin)–SELPLG (PSGL-1) axis to induce irreversible functional exhaustion of CD8^+^ T cells—a paradigm of CRL-mediated facilitation of profound immunosuppression (59). The upstream mechanism may involve CRL-mediated regulation of the secretion of key recruitment factors (e.g., IL-8 and CSF-1) by tumor cells or cancer-associated fibroblasts (CAFs).

##### 3.2.1.3 Mechanism 3: direct regulation of effector immune functions via vesicular communication

The immunomodulatory effects of CRLs can extend beyond the tumor cells via extracellular vesicles (EVs, such as exosomes), achieving long-distance intercellular communication. Tumor cells package specific CRLs (or their derived circular RNAs) into exosomes and deliver them to immune cells within the TIME. For example, HCC-derived exosomes can transport circCCAR1 to CD8^+^ T cells. circCCAR1 directly binds to and stabilizes the PD-1 protein, blocking the ubiquitin-mediated degradation pathway mediated by E3 ubiquitin ligases (e.g., CBL-B). This results in the aberrant accumulation of PD-1 on the T cell surface and sustained transmission of inhibitory signals, thereby driving T cell exhaustion (13). This vesicle-mediated delivery represents a strategy for the direct reprogramming of effector immune cell functions by tumor cells.

##### 3.2.1.4 Mechanism 4: synergistic upregulation of the immune checkpoint network

CRLs can concurrently upregulate the immune checkpoint network, constructing a multi-layered inhibitory signal barrier. In high-risk HCC patients defined by CRL signatures, the expression of multiple checkpoint molecules, including PD-L1, CTLA-4, LAG-3, and TIM-3, is significantly elevated (51). Paradoxically, this upregulation does not necessarily confer sensitivity to ICIs. Instead, the simultaneous overexpression of multiple inhibitory receptors signifies a state of “terminal T cell exhaustion” and entrenched immunological tolerance, rendering single-agent blockade ineffective (13, 59). This synergistic upregulation may be achieved by activating common oncogenic transcription factors (e.g., STAT3, NF-κB) or signaling pathways (e.g., PI3K/AKT). For instance, MKLN1-AS upregulates hepatoma-derived growth factor (HDGF) via a ceRNA mechanism, subsequently activating downstream pathways to promote PD-L1 transcription (55). Furthermore, while exosomal circCCAR1 stabilizes PD-1 on T cells, its host gene CCAR1 can also upregulate PD-L1 expression within tumor cells by enhancing the β-catenin/TCF4 signaling pathway, forming a “dual immunosuppressive lock” targeting both tumor cells and T cells (13).

#### 3.2.2 Functional heterogeneity: the dichotomy of resistance and sensitization

A critical, yet previously underexplored paradigm regarding CRL biology is the extent to which these molecules uniformly drive immunotherapy resistance. Accumulating evidence challenges the monolithic view of CRLs as solely immunosuppressive factors, revealing instead a functional dichotomy: while the majority of identified CRLs in HCC indeed facilitate immune evasion, a distinct subset possesses the capacity to remodel the tumor immune microenvironment (TIME) toward an immune-permissive state.

1. Mechanisms of Immune Exclusion: The “Brake” Effect Predominant oncogenic CRLs (e.g., MKLN1-AS and AL133243.2) effectively raise the threshold for cuproptosis by epigenetically or transcriptionally suppressing core metabolic enzymes, including FDX1 and DLAT (53, 54). This suppression not only ensures tumor cell survival under copper stress but also abrogates the release of Damage-Associated Molecular Patterns (DAMPs)—such as ATP, HMGB1, and calreticulin—which are obligate mediators of Immunogenic Cell Death (ICD) (41, 57). Consequently, the absence of these “danger signals” impedes dendritic cell (DC) maturation and antigen cross-presentation, ultimately leading to a “cold” tumor phenotype characterized by a paucity of infiltrating CD8+ cytotoxic T cells and an enrichment of immunosuppressive M2 macrophages (52, 53).
2. Mechanisms of Immune Sensitization: The “Accelerator” Effect Conversely, a subset of putative tumor-suppressive CRLs functions to potentiate antitumor immunity. A prime example is LINC02362, which is downregulated in HCC tissues. Mechanistically, LINC02362 acts as a competitive endogenous RNA (ceRNA) for miR-18a-5p, thereby relieving post-transcriptional repression of FDX1 (51). The restoration of FDX1 levels sensitizes tumor cells to copper-induced proteotoxic stress, facilitating robust ICD upon therapeutic stimulation. Crucially, this process transcends mere cytotoxicity; by releasing powerful adjuvant signals such as ATP and HMGB1, it effectively “heats up” the originally “cold” tumor immune microenvironment. This immunogenic shift triggers the secretion of tumor-associated antigens and pro-inflammatory cytokines (e.g., Type I Interferons), which theoretically reinvigorate the cGAS-STING signaling axis within the TIME (51). Thus, specific CRLs can convert the immunosuppressive milieu into an inflamed state, potentially enhancing the efficacy of immune checkpoint inhibitors (ICIs).

#### 3.2.3 Future challenges: deciphering complexity and context-dependence

Achieving a profound understanding of CRL functional heterogeneity faces substantial challenges, primarily stemming from the inherent complexity of tumor biology:

Intratumoral Heterogeneity: Within a single HCC tumor, distinct spatial regions or cellular subpopulations (e.g., tumor core vs. invasive margin, cancer stem cells vs. differentiated cells) may exhibit vastly different CRL expression profiles. The functions of these CRLs may differ fundamentally across malignant cells, immune cells (e.g., tumor-associated macrophages), and stromal cells (60).

Dynamic Evolution under Therapeutic Pressure: The expression profiles and functions of CRLs may undergo adaptive alterations under the selective pressure of ICI therapy. For instance, a CRL initially exerting an immune-sensitizing effect may shift to support resistance during treatment due to functional or regulatory network rewiring (7).

Network Superposition and Counteraction: A single CRL may simultaneously participate in multiple, potentially opposing signaling pathways. Its ultimate net biological effect is the result of the interplay between these pathways and is highly contingent upon the specific intracellular and extracellular context.

Summary

The mechanisms by which CRLs drive immunotherapy resistance in HCC constitute a stereoscopic network encompassing the regulation of intrinsic cell death sensitivity, long-distance intercellular communication, and the systemic modulation of immune checkpoint networks. However, the simplistic categorization of CRLs as mere “resistance drivers” no longer captures their full biological complexity. The field is currently at a pivotal transition point, moving from “correlative description” to “functional classification.” Future core mandates include leveraging cutting-edge technologies—such as single-cell multi-omics, spatial transcriptomics, CRISPR screens, and conditional cell-specific knockout models—to precisely elucidate the function of individual CRLs within specific cell types and microenvironmental contexts (41, 60). Establishing a causal logical chain linking “regulatory direction (pro-/anti-cuproptosis) — immune phenotype (pro-/anti-tumor immunity) — therapeutic response” is paramount. Only by achieving this precise functional decoding can we realize a paradigm shift from “broadly targeting CRLs” to “precisely targeting specific functional CRL subsets, “ thereby designing truly effective and personalized combination strategies to overcome HCC immunotherapy resistance.

### 3.3 Pan-cancer parallels: convergent immune microenvironment remodeling

Although this review focuses on the critical role of CRLs in HCC immunotherapy resistance, the “CRL–metabolic reprogramming–immune suppression” axis exhibits striking commonalities across other solid tumors (**Table 3**). While the specific lncRNAs may differ, their downstream effects on the tumor immune microenvironment (TIME) often converge on immune exclusion.

**Table 3:** Cross-cancer comparison of key verified CRLs: mechanisms and functional heterogeneity.

Cancer type	Representative CRL	Expression pattern	Core molecular mechanism	Role in cuproptosis	Immune/therapeutic implication	Ref.
HCC	LINC02362	Downregulated	Sponges miR-18a-5p to upregulate FDX1	Promoter (Sensitizer)	Enhances sensitivity to oxaliplatin and copper ionophores	(51)
HCC	MKLN1-AS	Upregulated	Sponges miR-654-3p to upregulate HDGF	Inhibitor (Resistor)	Promotes immune exclusion; drives progression	(54)
NSCLC	LINC01128	Upregulated	Sponges miR-576-5p to regulate CDKN3	Regulator	Associated with reduced CD8+ T cell infiltration	(61)

HCC, Hepatocellular Carcinoma; NSCLC, Non-Small Cell Lung Cancer; FDX1, Ferredoxin 1; HDGF, Hepatoma-Derived Growth Factor; CDKN3, Cyclin-dependent kinase inhibitor 3.

Convergent Remodeling in Lung and Breast Cancers In non-small cell lung cancer (NSCLC) and triple-negative breast cancer (TNBC), high-risk scores based on CRL signatures [such as models including AC026355.2 or USP2-AS1 (61, 62)] consistently map to “cold tumor” characteristics. Specifically, these signatures correlate with a significant reduction in CD8+ T cell infiltration accompanied by an increased abundance of regulatory T cells (Tregs) and M2-like macrophages. This indicates that regardless of the upstream lncRNA variation, the capacity of CRLs to drive immune evasion is a universal pathological feature shared by multiple cancer types.

#### 3.3.1 Mechanistic commonality: universality of ceRNA networks and immune suppression

Homogeneity in Immune Microenvironment Prediction: In various solid tumors, including HCC, NSCLC, and gastric cancer, multi-gene signatures based on CRLs robustly stratify patient prognosis. More importantly, high-risk scores consistently map to immunosuppressive microenvironmental features, such as M2 macrophage polarization, elevated Treg infiltration, and functional exhaustion of CD8^+^ T cells (63). This indicates that the function of CRLs as biomarkers indicating an “immune-cold” status possesses pan-cancer applicability.

The Central Regulatory Role of ceRNA Networks: A shared mechanistic principle across these cancers is the competitive endogenous RNA (ceRNA) network. Whether via the MKLN1-AS/miR-654-3p/HDGF axis in HCC (54) or the LINC01128/miR-576-5p/CDKN3 axis in NSCLC (61), CRLs consistently exert oncogenic functions by sequestering specific miRNAs to disrupt post-transcriptional regulatory networks. This suggests that while the specific lncRNAs differ by tissue, the “ceRNA-mediated immune evasion” represents a universal strategy utilized by CRLs to drive tumor progression.

#### 3.3.2 Tissue-specific distribution of key CRLs

Despite the functional convergence on immune suppression, distinct cancer types possess specific “core” CRLs. For example, AL133243.2 is a focal point of immune microenvironment regulation in HCC (52) but is rarely reported in other cancers; conversely, USP2-AS1, which is critical in breast cancer, is not a primary driver in HCC (62). This reflects the spatiotemporal specificity of lncRNA expression and the differential weighting of their roles across distinct tumor initiation and progression trajectories.

#### 3.3.3 Implications for future cross-cancer research

The comparative analysis reveals that CRL research relies on specific tumor histological contexts. Future studies should focus on the following strategic directions:

Systematically Charting a Pan-Cancer Regulatory Atlas of CRLs: Utilize multi-omics big data to laterally compare the expression regulatory networks and downstream signaling pathways across different cancer types, distinguishing between “housekeeping CRLs” and “tissue-specific CRLs.”

Exploring Personalized Therapeutic Strategies: For CRLs with high tissue specificity, develop precise companion diagnostic tools to realize personalized interventions based on molecular subtyping. By targeting the unique “core” CRLs of each cancer type (e.g., AL133243.2 for HCC), therapies can achieve maximum efficacy with minimal off-target effects.

## 4 Translational potential: from biomarkers to precision therapeutic strategies

The pivotal role of CRLs in HCC tumorigenesis and the remodeling of the tumor immune microenvironment renders them targets of significant clinical translational value. This section systematically delineates the clinical translational trajectory of CRL-based interventions across three dimensions: precision diagnosis, innovative therapies, and combinatorial strategies.

### 4.1 CRLs as composite biomarkers for precision diagnosis and treatment

Given the marked molecular heterogeneity of HCC, the predictive efficacy of single molecules is often limited. Developing composite biomarker systems based on CRLs is key to achieving precise patient stratification and therapeutic monitoring.

Risk Stratification and Therapeutic Decision-Making: Existing prognostic models (e.g., the CRDELSig signature) have been confirmed to effectively discriminate patient survival risks (23, 52). Future translational directions lie in the multidimensional integration of CRL scores with tumor mutational burden (TMB), microsatellite instability (MSI), and immune checkpoint expression levels. Such integrated models can not only identify “high-risk populations” with poor prognosis but also precisely screen for potential dominant populations capable of benefiting from combination therapy comprising “cuproptosis inducers + ICIs” (38, 47).

Liquid Biopsy and Dynamic Monitoring of Resistance: Compared to the invasiveness and lag of tissue biopsy, exosome-encapsulated lncRNAs exhibit extremely high stability in the circulatory system (12). Clinical applications should focus on developing high-sensitivity detection panels based on droplet digital PCR (ddPCR) to monitor the dynamic changes of specific CRLs (e.g., LINC02362 or MKLN1-AS) in the blood. During treatment, abnormal fluctuations in circulating CRL levels may precede radiological progression, thereby serving as early warning signals for acquired resistance to ICIs and guiding the timely adjustment of therapeutic regimens (64, 65).

### 4.2 Targeted therapeutic strategies: from molecular interference to systemic reprogramming

Targeting the CRL-driven immune resistance network, therapeutic strategies can be categorized into “direct targeting” and “indirect modulation, “ aiming to restore cuproptosis sensitivity and reshape the immune microenvironment.

#### 4.2.1 Direct strategies: precision silencing of oncogenic CRLs directly targeting resistance-driving lncRNAs is the fundamental pathway to abrogate their oncogenic functions

Antisense Oligonucleotides (ASOs) and Chemical Modification Technologies: ASOs specifically silence target RNA via RNase H-mediated degradation mechanisms. For HCC, the utilization of N-acetylgalactosamine (GalNAc) conjugation technology is key to breaking through delivery bottlenecks. GalNAc ligands can bind with high affinity to the asialoglycoprotein receptor (ASGPR) on the surface of hepatocytes, enabling the liver-specific uptake of ASOs. This not only drastically improves gene silencing efficiency but also effectively avoids off-target toxicity in the kidney and nervous system (66).

Small Molecule Inhibitors Interfering with Protein-Protein Interactions (PPI): Many CRLs function by recruiting specific proteins (e.g., FDX1). Developing small molecule compounds capable of occupying specific binding pockets in the secondary structure of lncRNAs to block the lncRNA-protein interaction interface represents a highly promising non-nucleic acid drug strategy (67).

CRISPR/Cas13 Editing Technology: Although still in the preclinical stage, RNA editing systems based on CRISPR/Cas13 offer higher specificity and efficiency than RNAi for targeting nuclear lncRNAs. Utilizing the Cas13d effector protein to specifically degrade oncogenic CRLs may become a disruptive technology for conquering “undruggable” lncRNA targets in the future (68).

#### 4.2.2 Indirect strategies: pharmacological modulation and synergistic combination effects mere induction of cuproptosis may face adaptive resistance from tumors; therefore, “combinatorial” strategies are the core of clinical translation

“Prime and Kill” Strategy: Utilizing copper ionophores (e.g., Elesclomol) as “primers.” They not only induce cuproptosis in tumor cells but, more critically, trigger immunogenic cell death (ICD), releasing danger signals such as ATP and HMGB1 (38, 41).This immunogenic burst converts the originally immunosuppressed “cold tumor” into an inflamed “hot tumor, “ thereby significantly elevating the response rate of subsequent PD-1/PD-L1 inhibitors (“killers”) (15, 30).

Copper Chelators for Improving Hypoxia and Vascular Normalization: For HCC subtypes with high copper burden and active angiogenesis, the use of copper chelators (e.g., tetrathiomolybdate, TTM) can reduce bioavailable copper levels. The dual benefit of this strategy lies in: on one hand, reducing extracellular matrix cross-linking by inhibiting LOX enzyme activity to soften the tumor stroma; and on the other hand, inhibiting the VEGF pathway to promote vascular normalization, thereby breaking physical barriers and facilitating the deep infiltration of effector T cells into the tumor core (45, 69).

#### 4.2.3 Advanced delivery systems: empowerment by intelligent nanomedicine nanotechnology not only resolves the solubility issues of hydrophobic drugs but also realizes spatiotemporally responsive release within the tumor microenvironment

Balance between Stimuli-Responsiveness and Safety: Designing intelligent nanocarriers sensitive to tumor microenvironmental characteristics (acidic pH, high ROS, high GSH) is key to reducing systemic copper toxicity (**Table 4**). For example, ROS-responsive linkers ensure that copper ionophores are released only within tumor cells with high oxidative stress levels, while remaining stable in normal liver tissue (25, 46).

**Table 4:** Representative nanodelivery systems developed for targeting cuproptosis.

Nano-system composition	Targeting strategy	Mechanism of action	Advantages
Polyethylene glycol-capped copper(I) oxide nanocomposite (PEG@Cu_2_O-ES) (25)	EPR effect & near-infrared photothermal response	Releases Elesclomol and Cu_2_O; photothermal effect enhances copper release and inhibits ATP-copper pump; induces copper-mediated cell death and sensitizes anti-PD-1 therapy	Synergistic photothermal therapy and immune reprogramming
Platelet-Encapsulated Cu_2_O/TBP-2 System (PTC) (70)	Platelet membrane-targeted tumor delivery	Acidic-conditioned Cu^+^ release; light-induced ROS depletion of GSH, blocking copper efflux; induces potent copper-mediated death and inhibits lung metastasis	High biocompatibility with prolonged circulation and immune memory activation
Macrophage Membrane-Coated Cu@ZIF-8 Sonosensitizer System (SonoCu) (46)	Macrophage membrane integrin targeting	Synergistic copper-mediated cell death with sonodynamic therapy; alleviates tumor hypoxia; depletes GSH and induces mitochondrial dysfunction	Hypoxia improvement and multi-pathway cell death induction
Copper-doped Au@MSN nanoplatform loaded with DSF (Au@MSN-Cu/PEG/DSF) (46)	EPR effect & near-infrared light-controlled release	Photothermal triggering releases DSF and Cu²^+^, generating CuET *in situ*; induces Cuproptosis and apoptosis, synergizing with photothermal therapy	Precise controlled release with minimal damage to normal tissues
Polymer-Coated Nanoparticles Encapsulating ES and Copper (NP@ES-Cu) (46)	ROS-Responsive Release	Releases ES and Cu in high-ROS tumor microenvironments, inducing mitochondrial copper accumulation and copper-induced cell death; synergizes with αPD-L1 for enhanced immunotherapy	Suitable for immunosuppressive tumor microenvironments

Dual Immune-Metabolic Reprogramming: Next-generation nanomedicines should pursue a “killing two birds with one stone” effect. For example, designing nanoplatforms that co-deliver cuproptosis inducers and STING pathway agonists. This design leverages mitochondrial DNA leakage generated by cuproptosis to synergistically activate the cGAS-STING pathway, triggering a potent Type I interferon response, thereby achieving synchronous activation of innate and adaptive immunity (70).

## 5 Discussion and future perspectives

### 5.1 The double-edged sword: critical insights into the dual immunological roles of cuproptosis

While current research enthusiastically positions cuproptosis as a novel vulnerability in hepatocellular carcinoma (HCC), a critical examination of the literature reveals a complex “double-edged sword” effect regarding its impact on antitumor immunity. Understanding this paradox—where copper signals can both ignite and extinguish immune responses—is pivotal for rational therapeutic design.

#### 5.1.1 The “accelerator”: promoting immunogenicity via mitochondrial stress

On one hand, cuproptosis functions as a potent immunogenic driver. Unlike apoptosis, which is often immunologically silent, cuproptosis triggered by copper ionophores (e.g., elesclomol) induces profound mitochondrial proteotoxic stress and membrane rupture (14, 41). This catastrophe triggers the release of Damage-Associated Molecular Patterns (DAMPs), including ATP, HMGB1, and mitochondrial DNA (mtDNA) (38). These danger signals are sensed by dendritic cells (DCs) via the cGAS-STING pathway or Toll-like receptors, facilitating DC maturation and the cross-priming of cytotoxic CD8+ T cells (17, 38). In this context, inducing cuproptosis effectively converts the “cold” immunosuppressive tumor microenvironment (TME) of HCC into an inflamed, “hot” state, overcoming the antigen-presentation defects typical of liver cancer.

#### 5.1.2 The “brake”: copper-driven immune evasion and checkpoint upregulation

Conversely, a growing body of evidence suggests that copper metabolism intrinsically supports immune evasion mechanisms. A seminal study by Voli et al. demonstrated that intratumoral copper levels act as a metabolic rheostat for PD-L1 expression (71). Mechanistically, copper ions bind to the CTR1 transporter and facilitate the phosphorylation of EGFR and STAT3, which transcriptionally upregulate PD-L1; simultaneously, copper promotes the ubiquitination inhibition of PD-L1, enhancing its protein stability. Consequently, sub-lethal copper accumulation—or the copper-rich environment characteristic of HCC—may paradoxically protect tumor cells from T cell killing by fortifying the PD-1/PD-L1 inhibitory axis (45). Furthermore, copper acts as a cofactor for enzymes that facilitate angiogenesis (e.g., lysyl oxidase), potentially fostering a hypoxic barrier that excludes T cell infiltration (14, 44).

#### 5.1.3 Critical synthesis: the threshold hypothesis and combination necessity

This dichotomy presents a strategic dilemma: while acute, lethal doses of copper induce immunogenic death, chronic, sub-lethal copper elevation may drive adaptive immune resistance. We propose a “Threshold Hypothesis”: the immunological outcome of copper targeting depends critically on crossing a lethality threshold that overwhelms the tumor’s adaptive PD-L1 upregulation. In the specific context of HCC, where the liver is the central organ for copper homeostasis and tumors often exhibit baseline copper overload, the risk of copper-induced immune suppression is particularly high. Therefore, relying solely on cuproptosis induction is likely insufficient and may even select for resistant clones with high PD-L1 expression. This mechanistic insight provides a compelling rationale for the obligatory combination of cuproptosis inducers with immune checkpoint inhibitors (ICIs). By using ICIs to block the “brake” (PD-L1) reinforced by copper, and copper ionophores to press the “accelerator” (ICD), clinicians may achieve a synergistic therapeutic efficacy that neither monotherapy can provide (15, 16).

### 5.2 Navigating the chasm: key challenges in clinical translation

Beyond the immunological paradox discussed above, although targeting cuproptosis-associated lncRNAs represents a conceptually novel strategy to overcome immunotherapy resistance in hepatocellular carcinoma, its translation into clinical practice faces significant challenges. A clear-eyed assessment of these hurdles is essential to chart a productive course for future research.

#### 5.2.1 Mechanistic complexity and tumor heterogeneity

Effective induction of cuproptosis relies heavily on intact mitochondrial respiratory function. This dependency, however, faces a major obstacle in the pronounced intra- and inter-tumor heterogeneity characteristic of HCC (60).HCC cells of different molecular subtypes exhibit markedly divergent sensitivities to cuproptosis inducers. For instance, ARID1A-deficient HCC cells, constrained by glycolysis, depend more heavily on mitochondrial respiration and thus show heightened sensitivity to copper ionophores. Conversely, cells with TCA cycle dysfunction or electron transport chain impairments readily develop resistance (14). This metabolic heterogeneity underscores the need for future research to leverage single-cell multi-omics technologies. These approaches can precisely map CRLs and cuproptosis pathway-specific regulatory networks at single-cell resolution across tumor, immune, and stromal cells. Building on this foundation, establishing a precision classification system based on molecular profiles and mitochondrial functional states is a prerequisite for implementing effective targeted therapies.

#### 5.2.2 Target specificity and therapeutic toxicity

A central concern for clinical translation is the risk of off-target toxicity caused by systemic perturbation of copper homeostasis. Since copper is an essential trace element, significant fluctuations in systemic levels can induce severe adverse effects, including hepatotoxicity, nephrotoxicity, and neurological impairment (15).To overcome this bottleneck, emerging nanodelivery systems offer distinct advantages. For instance, platelet-mimetic PTC systems achieve tumor enrichment through natural targeting, while macrophage-coated SonoCu systems utilize integrin-mediated active targeting (46).A promising future direction involves developing next-generation smart nanocarriers that respond to specific tumor microenvironment signals (e.g., pH, glutathione levels, or enzyme activity). Such systems would enable precise, controlled drug release, enhancing therapeutic efficacy while minimizing systemic toxicity.

#### 5.2.3 Lag in biomarker development

A significant gap exists in the availability of validated clinical biomarkers for predicting tumor susceptibility to cuproptosis (72). Although prognostic models based on cuproptosis-related genes and CRLs show promise in retrospective analyses, their generalizability across diverse populations and HCC subtypes requires large-scale validation (15). Progress in this area hinges on the systematic validation and optimization of these predictive models using large prospective cohorts, followed by their translation into clinically applicable decision-making tools. Concurrently, non-invasive dynamic monitoring technologies should be actively developed (73), Examples include employing ^64^Cu-PET imaging to visualize *in vivo* copper distribution (74, 75)and developing liquid biopsy assays for detecting extracellular CRLs in blood (64), These approaches could provide objective data for real-time treatment adjustment.

#### 5.2.4 Limited clinical translational evidence

It must be acknowledged that most cuproptosis-related research remains confined to the preclinical stage, with no clinical trials conducted for HCC to date. Early clinical trials in other cancer types have yielded inconsistent results, underscoring the challenges in translation. For instance, a Phase III trial of Elesclomol combined with paclitaxel in melanoma was prematurely terminated due to a lack of survival benefit and increased toxicity; similarly, trials in relapsed acute myeloid leukemia failed to demonstrate significant efficacy (15).These setbacks highlight deficiencies in drug delivery, pharmacokinetics, and, crucially, patient selection strategies. Consequently, future clinical translation efforts must be strategically designed. Initial Phase I/II trials should be conducted in biomarker-enriched populations, prioritizing rational combination regimens. Given the close interaction between cuproptosis and the immune microenvironment, a triple therapy approach combining cuproptosis inducers, immune checkpoint inhibitors, and anti-angiogenic drugs holds particular synergistic potential (69). Additionally, exploring optimization strategies like pulsed or sequential dosing is crucial for preventing or overcoming adaptive resistance (76).

### 5.3 Future outlook

Notwithstanding these challenges, the field is poised for advancement. Deepening insights into CRL regulatory networks, coupled with the convergence of nanotechnology, rational combination therapies, and multi-omics-based precision profiling, sustain the promise of targeting the CRL-cuproptosis axis as a viable, albeit complex, strategy to overcome immunotherapy resistance in HCC. By systematically addressing the core challenges outlined herein, the research community can work toward achieving substantial breakthroughs—transitioning from theoretical exploration to clinical impact—within the next decade, ultimately offering new therapeutic hope for patients with hepatocellular carcinoma.

## 6 Conclusion

In conclusion, the establishment of immunological tolerance within the tumor microenvironment constitutes a primary impediment to the efficacy of immune checkpoint inhibitors in hepatocellular carcinoma (HCC). This review elucidates the critical function of copper death-related lncRNAs (CRLs) as epigenetic architects of this tolerogenic state. By orchestrating a multifaceted regulatory network—encompassing the repression of cuproptosis-induced immunogenicity, the exosome-mediated propagation of suppressive signals, and the synergistic upregulation of co-inhibitory checkpoints—CRLs actively foster immune exclusion and T cell exhaustion. Significantly, the identification of functional heterogeneity among CRLs unveils a therapeutic dichotomy: while oncogenic CRLs reinforce tolerance, tumor-suppressive CRLs possess the capacity to abrogate tolerance by triggering immunogenic cell death (ICD). From a translational perspective, the development of CRL-based composite biomarkers and stimulus-responsive nanodelivery systems offers a precision medicine approach to dynamically monitor and modulate these metabolic-immune interactions. Ultimately, targeting the CRL-cuproptosis axis represents a transformative therapeutic strategy to dismantle tumor-induced tolerance, reinstate immune surveillance, and substantially improve clinical outcomes in patients with HCC.

## References

[1] Rumgay H Arnold M Ferlay J Lesi O Cabasag CJ Vignat J . Global burden of primary liver cancer in 2020 and predictions to 2040. J Hepatol. (2022) 77:1598–606. doi: 10.1016/j.jhep.2022.08.021, PMID: 36208844 PMC9670241

[2] Chan SL Sun H-C Xu Y Zeng H El-Serag HB Lee JM . The Lancet Commission on addressing the global hepatocellular carcinoma burden: comprehensive strategies from prevention to treatment. Lancet. (2025) 406:731–78. doi: 10.1016/S0140-6736(25)01042-6, PMID: 40744051

[3] Forner A Reig M Bruix J . Hepatocellular carcinoma. Lancet. (2018) 391:1301–14. doi: 10.1016/S0140-6736(18)30010-2, PMID: 29307467

[4] Cheng K Cai N Zhu J Yang X Liang H Zhang W . Tumor-associated macrophages in liver cancer: From mechanisms to therapy. Cancer Commun. (2022) 42:1112–40. doi: 10.1002/cac2.12345, PMID: 36069342 PMC9648394

[5] Kalasekar SM Garrido-Laguna I Evason KJ . Immune checkpoint inhibitors in combinations for hepatocellular carcinoma. Hepatology. (2021) 73:2591–3. doi: 10.1002/hep.31706, PMID: 33434363

[6] Oura K Morishita A Tani J Masaki T . Tumor immune microenvironment and immunosuppressive therapy in hepatocellular carcinoma: a review. Int J Mol Sci. (2021) 22:5801. doi: 10.3390/ijms22115801, PMID: 34071550 PMC8198390

[7] Ladd AD Duarte S Sahin I Zarrinpar A . Mechanisms of drug resistance in HCC. Hepatology. (2024) 79:926–40. doi: 10.1097/HEP.0000000000000237, PMID: 36680397

[8] Rimassa L Finn RS Sangro B . Combination immunotherapy for hepatocellular carcinoma. J Hepatol. (2023) 79:506–15. doi: 10.1016/j.jhep.2023.03.003, PMID: 36933770

[9] Sangro B Sarobe P Hervás-Stubbs S Melero I . Advances in immunotherapy for hepatocellular carcinoma. Nat Rev Gastroenterol Hepatol. (2021) 18:525–43. doi: 10.1038/s41575-021-00438-0, PMID: 33850328 PMC8042636

[10] Cheng A-L Qin S Ikeda M Galle PR Ducreux M Kim T-Y . Updated efficacy and safety data from IMbrave150: Atezolizumab plus bevacizumab vs. sorafenib for unresectable hepatocellular carcinoma. J Hepatol. (2022) 76:862–73. doi: 10.1016/j.jhep.2021.11.030, PMID: 34902530

[11] Hao L Li S Ye F Wang H Zhong Y Zhang X . The current status and future of targeted-immune combination for hepatocellular carcinoma. Front Immunol. (2024) 15:1418965. doi: 10.3389/fimmu.2024.1418965, PMID: 39161764 PMC11330771

[12] Xu Z Chen Y Ma L Chen Y Liu J Guo Y . Role of exosomal non-coding RNAs from tumor cells and tumor-associated macrophages in the tumor microenvironment. Mol Ther. (2022) 30:3133–54. doi: 10.1016/j.ymthe.2022.01.046, PMID: 35405312 PMC9552915

[13] Hu Z Chen G Zhao Y Gao H Li L Yin Y . Exosome-derived circCCAR1 promotes CD8 + T-cell dysfunction and anti-PD1 resistance in hepatocellular carcinoma. Mol Cancer. (2023) 22:55. doi: 10.1186/s12943-023-01759-1, PMID: 36932387 PMC10024440

[14] Tang D Kroemer G Kang R . Targeting cuproplasia and cuproptosis in cancer. Nat Rev Clin Oncol. (2024) 21:370–88. doi: 10.1038/s41571-024-00876-0, PMID: 38486054

[15] Zhou Y-F Zhu Y-W Hao M-Y Li H-J Han H-S Li Y-G . Targeting cuproptosis in liver cancer: Molecular mechanisms and therapeutic implications. Apoptosis. (2025) 30:2163–90. doi: 10.1007/s10495-025-02150-9, PMID: 40775595

[16] Boaru DL Leon-Oliva DD Castro-Martinez PD Garcia-Montero C Fraile-Martinez O García-González B . Cuproptosis: Current insights into its multifaceted role in disease, cancer, and translational/therapeutic opportunities. Biomedicine Pharmacotherapy. (2025) 190:118422. doi: 10.1016/j.biopha.2025.118422, PMID: 40774019

[17] Zhang S Huang Q Ji T Li Q Hu C . Copper homeostasis and copper-induced cell death in tumor immunity: implications for therapeutic strategies in cancer immunotherapy. biomark Res. (2024) 12:130. doi: 10.1186/s40364-024-00677-8, PMID: 39482784 PMC11529036

[18] Zhang X Tang B Luo J Yang Y Weng Q Fang S . Cuproptosis, ferroptosis and PANoptosis in tumor immune microenvironment remodeling and immunotherapy: culprits or new hope. Mol Cancer. (2024) 23:255. doi: 10.1186/s12943-024-02130-8, PMID: 39543600 PMC11566504

[19] Statello L Guo C-J Chen L-L Huarte M . Gene regulation by long non-coding RNAs and its biological functions. Nat Rev Mol Cell Biol. (2021) 22:96–118. doi: 10.1038/s41580-020-00315-9, PMID: 33353982 PMC7754182

[20] Shi X Sun M Liu H Yao Y Song Y . Long non-coding RNAs: A new frontier in the study of human diseases. Cancer Lett. (2013) 339:159–66. doi: 10.1016/j.canlet.2013.06.013, PMID: 23791884

[21] Wu Z Liu X Liu L Deng H Zhang J Xu Q . Regulation of lncRNA expression. Cell Mol Biol Lett. (2014) 19:561–75. doi: 10.2478/s11658-014-0212-6, PMID: 25311814 PMC6275606

[22] Yang J Liu F Wang Y Qu L Lin A . LncRNAs in tumor metabolic reprogramming and immune microenvironment remodeling. Cancer Lett. (2022) 543:215798. doi: 10.1016/j.canlet.2022.215798, PMID: 35738332

[23] Chen S Liu P Zhao L Han P Liu J Yang H . A novel cuproptosis-related prognostic lncRNA signature for predicting immune and drug therapy response in hepatocellular carcinoma. Front Immunol. (2022) 13:954653. doi: 10.3389/fimmu.2022.954653, PMID: 36189204 PMC9521313

[24] Liu Y Jiang J . A novel cuproptosis-related lncRNA signature predicts the prognosis and immunotherapy for hepatocellular carcinoma. Cancer Bio. (2023) 37:13–26. doi: 10.3233/CBM-220259, PMID: 37005878 PMC12412818

[25] Meena R Sahoo SS Sunil A Manna D . Cuproptosis: A copper-mediated programmed cell death. Chem Asian J. (2025) 20:e202400934. doi: 10.1002/asia.202400934, PMID: 39520466

[26] Tong X Tang R Xiao M Xu J Wang W Zhang B . Targeting cell death pathways for cancer therapy: recent developments in necroptosis, pyroptosis, ferroptosis, and cuproptosis research. J Hematol Oncol. (2022) 15:174. doi: 10.1186/s13045-022-01392-3, PMID: 36482419 PMC9733270

[27] Mou Y Wang J Wu J He D Zhang C Duan C . Ferroptosis, a new form of cell death: opportunities and challenges in cancer. J Hematol Oncol. (2019) 12:34. doi: 10.1186/s13045-019-0720-y, PMID: 30925886 PMC6441206

[28] Zhang C Liu X Jin S Chen Y Guo R . Ferroptosis in cancer therapy: a novel approach to reversing drug resistance. Mol Cancer. (2022) 21:47. doi: 10.1186/s12943-022-01530-y, PMID: 35151318 PMC8840702

[29] Moyer A Tanaka K Cheng EH . Apoptosis in cancer biology and therapy. Annu Rev Pathol. (2025) 20:303–28. doi: 10.1146/annurev-pathmechdis-051222-115023, PMID: 39854189

[30] Zhou S Liu J Wan A Zhang Y Qi X . Epigenetic regulation of diverse cell death modalities in cancer: a focus on pyroptosis, ferroptosis, cuproptosis, and disulfidptosis. J Hematol Oncol. (2024) 17:22. doi: 10.1186/s13045-024-01545-6, PMID: 38654314 PMC11040947

[31] Pasparakis M Vandenabeele P . Necroptosis and its role in inflammation. Nature. (2015) 517:311–20. doi: 10.1038/nature14191, PMID: 25592536

[32] Galluzzi L Vitale I Aaronson SA Abrams JM Adam D Agostinis P . Molecular mechanisms of cell death: recommendations of the Nomenclature Committee on Cell Death 2018. Cell Death Differ. (2018) 25:486–541. doi: 10.1038/s41418-017-0012-4, PMID: 29362479 PMC5864239

[33] Mizushima N Komatsu M . Autophagy: renovation of cells and tissues. Cell. (2011) 147:728–41. doi: 10.1016/j.cell.2011.10.026, PMID: 22078875

[34] Klionsky DJ Abdel-Aziz AK Abdelfatah S Abdellatif M Abdoli A Abel S . Guidelines for the use and interpretation of assays for monitoring autophagy (4th edition)^1^. Autophagy. (2021) 17:1–382. doi: 10.1080/15548627.2020.1797280, PMID: 33634751 PMC7996087

[35] Liu W Lin W Yan L Xu W Yang J . Copper homeostasis and cuproptosis in cancer immunity and therapy. Immunol Rev. (2024) 321:211–27. doi: 10.1111/imr.13276, PMID: 37715546

[36] Chen L Min J Wang F . Copper homeostasis and cuproptosis in health and disease. Sig Transduct Target Ther. (2022) 7:378. doi: 10.1038/s41392-022-01229-y, PMID: 36414625 PMC9681860

[37] Shao K Shen H Chen X Shao Z Liu Y Wang Y . Copper transporter gene ATP7A: A predictive biomarker for immunotherapy and targeted therapy in hepatocellular carcinoma. Int Immunopharmacol. (2023) 114:109518. doi: 10.1016/j.intimp.2022.109518, PMID: 36502594

[38] Zhang L Xie A Ma J Liu H Zeng C . Unveiling Cuproptosis: Mechanistic insights, roles, and leading advances in oncology. Biochim Biophys Acta Rev Cancer. (2024) 1879:189180. doi: 10.1016/j.bbcan.2024.189180, PMID: 39276875

[39] Zhang C Huang T Li L . Targeting cuproptosis for cancer therapy: mechanistic insights and clinical perspectives. J Hematol Oncol. (2024) 17:68. doi: 10.1186/s13045-024-01589-8, PMID: 39152464 PMC11328505

[40] Sun B Ding P Song Y Zhou J Chen X Peng C . FDX1 downregulation activates mitophagy and the PI3K/AKT signaling pathway to promote hepatocellular carcinoma progression by inducing ROS production. Redox Biol. (2024) 75:103302. doi: 10.1016/j.redox.2024.103302, PMID: 39128228 PMC11366913

[41] Tsvetkov P Coy S Petrova B Dreishpoon M Verma A Abdusamad M . Copper induces cell death by targeting lipoylated TCA cycle proteins. Science. (2022) 375:1254–61. doi: 10.1126/science.abf0529, PMID: 35298263 PMC9273333

[42] Tian Z Jiang S Zhou J Zhang W . Copper homeostasis and cuproptosis in mitochondria. Life Sci. (2023) 334:122223. doi: 10.1016/j.lfs.2023.122223, PMID: 38084674

[43] Li Z Zhou H Zhai X Gao L Yang M An B . MELK promotes HCC carcinogenesis through modulating cuproptosis-related gene DLAT-mediated mitochondrial function. Cell Death Dis. (2023) 14:733. doi: 10.1038/s41419-023-06264-3, PMID: 37949877 PMC10638394

[44] Zhang R Tan Y Xu K Huang N Wang J Liu M . Cuproplasia and cuproptosis in hepatocellular carcinoma: mechanisms, relationship and potential role in tumor microenvironment and treatment. Cancer Cell Int. (2025) 25:137. doi: 10.1186/s12935-025-03683-4, PMID: 40205387 PMC11983883

[45] Feng Q Huo C Wang M Huang H Zheng X Xie M . Research progress on cuproptosis in cancer. Front Pharmacol. (2024) 15:1290592. doi: 10.3389/fphar.2024.1290592, PMID: 38357312 PMC10864558

[46] Lu J Miao Y Li Y . Cuproptosis: advances in stimulus-responsive nanomaterials for cancer therapy. Adv Healthc Mater. (2024) 13:2400652. doi: 10.1002/adhm.202400652, PMID: 38622782

[47] Lu D Liao J Cheng H Ma Q Wu F Xie F . Construction and systematic evaluation of a machine learning-based cuproptosis-related lncRNA score signature to predict the response to immunotherapy in hepatocellular carcinoma. Front Immunol. (2023) 14:1097075. doi: 10.3389/fimmu.2023.1097075, PMID: 36761763 PMC9905126

[48] Li D Jin S Chen P Zhang Y Li Y Zhong C . Comprehensive analysis of cuproptosis-related lncRNAs for prognostic significance and immune microenvironment characterization in hepatocellular carcinoma. Front Immunol. (2023) 13:991604. doi: 10.3389/fimmu.2022.991604, PMID: 36685508 PMC9846072

[49] Chen Y Tang L Huang W Abisola FH Zhang Y Zhang G . Identification of a prognostic cuproptosis-related signature in hepatocellular carcinoma. Biol Direct. (2023) 18:4. doi: 10.1186/s13062-023-00358-w, PMID: 36750831 PMC9903524

[50] Wang Y Zhang Y Wang L Zhang N Xu W Zhou J . Development and experimental verification of a prognosis model for cuproptosis-related subtypes in HCC. Hepatol Int. (2022) 16:1435–47. doi: 10.1007/s12072-022-10381-0, PMID: 36065073

[51] Quan B Liu W Yao F Li M Tang B Li J . LINC02362/hsa-miR-18a-5p/FDX1 axis suppresses proliferation and drives cuproptosis and oxaliplatin sensitivity of hepatocellular carcinoma. Am J Cancer Res. (2023) 13:5590–609. PMC10695789 38058825

[52] Yuan W Xiao J Zhang J Mao B Wang P Wang B . Identification of a cuproptosis and copper metabolism gene–related lncRNAs prognostic signature associated with clinical and immunological characteristics of hepatocellular carcinoma. Front Oncol. (2023) 13:1153353. doi: 10.3389/fonc.2023.1153353, PMID: 37056336 PMC10086263

[53] Liu C Wu S Lai L Liu J Guo Z Ye Z . Comprehensive analysis of cuproptosis-related lncRNAs in immune infiltration and prognosis in hepatocellular carcinoma. BMC Bioinf. (2023) 24:4. doi: 10.1186/s12859-022-05091-1, PMID: 36597032 PMC9811804

[54] Gao W Chen X Chi W Xue M . Long non−coding RNA MKLN1−AS aggravates hepatocellular carcinoma progression by functioning as a molecular sponge for miR−654−3p, thereby promoting hepatoma−derived growth factor expression. Int J Mol Med. (2020) 46:1743–54. doi: 10.3892/ijmm.2020.4722, PMID: 33000222 PMC7521589

[55] Song D Wang X Wang Y Liang W Luo J Zheng J . Integrated analysis of N1-methyladenosine methylation regulators-related lncRNAs in hepatocellular carcinoma. Cancers. (2023) 15:1800. doi: 10.3390/cancers15061800, PMID: 36980686 PMC10046959

[56] Xu L Chen S Li Q Chen X Xu Y Zhou Y . Integrating bioinformatics and experimental validation to unveil disulfidptosis-related lncRNAs as prognostic biomarker and therapeutic target in hepatocellular carcinoma. Cancer Cell Int. (2024) 24:30. doi: 10.1186/s12935-023-03208-x, PMID: 38218909 PMC10788009

[57] Chen X Sun M Feng W Chen J Ji X Xie M . An integrative analysis revealing cuproptosis-related lncRNAs signature as a novel prognostic biomarker in hepatocellular carcinoma. Front Genet. (2023) 14:1056000. doi: 10.3389/fgene.2023.1056000, PMID: 36845390 PMC9950118

[58] Guo C Zhou S Yi W Yang P Li O Liu J . Long non-coding RNA muskelin 1 antisense RNA (MKLN1-AS) is a potential diagnostic and prognostic biomarker and therapeutic target for hepatocellular carcinoma. Exp Mol Pathol. (2021) 120:104638. doi: 10.1016/j.yexmp.2021.104638, PMID: 33878313

[59] Meng Y Ye F Nie P Zhao Q An L Wang W . Immunosuppressive CD10+ALPL+ neutrophils promote resistance to anti-PD-1 therapy in HCC by mediating irreversible exhaustion of T cells. J Hepatol. (2023) 79:1435–49. doi: 10.1016/j.jhep.2023.08.024, PMID: 37689322

[60] Ho DW-H Tsui Y-M Chan L-K Sze KM-F Zhang X Cheu JW-S . Single-cell RNA sequencing shows the immunosuppressive landscape and tumor heterogeneity of HBV-associated hepatocellular carcinoma. Nat Commun. (2021) 12:3684. doi: 10.1038/s41467-021-24010-1, PMID: 34140495 PMC8211687

[61] Lu L Qin Y Wang M Deng Y Lin Y . A 4−cuproptosis−related lncRNA theragnostic signature predicts survival and immunotherapy response in patients with lung adenocarcinoma. Mol Clin Oncol. (2025) 24:1–13. doi: 10.3892/mco.2025.2912, PMID: 41220401 PMC12598532

[62] Xu Q-T Wang Z-W Cai M-Y Wei J-F Ding Q . A novel cuproptosis-related prognostic 2-lncRNAs signature in breast cancer. Front Pharmacol. (2023) 13:1115608. doi: 10.3389/fphar.2022.1115608, PMID: 36699089 PMC9868634

[63] Feng A He L Chen T Xu M . A novel cuproptosis-related lncRNA nomogram to improve the prognosis prediction of gastric cancer. Front Oncol. (2022) 12:957966. doi: 10.3389/fonc.2022.957966, PMID: 36106123 PMC9465020

[64] Guo T Tang X-H Gao X-Y Zhou Y Jin B Deng Z-Q . A liquid biopsy signature of circulating exosome-derived mRNAs, miRNAs and lncRNAs predict therapeutic efficacy to neoadjuvant chemotherapy in patients with advanced gastric cancer. Mol Cancer. (2022) 21:216. doi: 10.1186/s12943-022-01684-9, PMID: 36510184 PMC9743536

[65] Park J Lee Y-T Agopian VG Liu JS Koltsova EK You S . Liquid biopsy in hepatocellular carcinoma: Challenges, advances, and clinical implications. Clin Mol Hepatol. (2025) 31:S255–84. doi: 10.3350/cmh.2024.0541, PMID: 39604328 PMC11925447

[66] Debacker AJ Voutila J Catley M Blakey D Habib N . Delivery of oligonucleotides to the liver with galNAc: from research to registered therapeutic drug. Mol Ther. (2020) 28:1759–71. doi: 10.1016/j.ymthe.2020.06.015, PMID: 32592692 PMC7403466

[67] Childs-Disney JL Yang X Gibaut QMR Tong Y Batey RT Disney MD . Targeting RNA structures with small molecules. Nat Rev Drug Discov. (2022) 21:736–62. doi: 10.1038/s41573-022-00521-4, PMID: 35941229 PMC9360655

[68] Abudayyeh OO Gootenberg JS Essletzbichler P Han S Joung J Belanto JJ . RNA targeting with CRISPR–cas13. Nature. (2017) 550:280–4. doi: 10.1038/nature24049, PMID: 28976959 PMC5706658

[69] Sequera C Maina F . Two is better than one: combinatorial receptor targeting enhances hepatocellular carcinoma (HCC) therapeutic response. Hepatobiliary Surg Nutr. (2022) 11:139–42. doi: 10.21037/hbsn-21-517, PMID: 35284507 PMC8847867

[70] Li L Zhou H Zhang C . Cuproptosis in cancer: biological implications and therapeutic opportunities. Cell Mol Biol Lett. (2024) 29:91. doi: 10.1186/s11658-024-00608-3, PMID: 38918694 PMC11201306

[71] Voli F Valli E Lerra L Kimpton K Saletta F Giorgi FM . Intratumoral copper modulates PD-L1 expression and influences tumor immune evasion. Cancer Res. (2020) 80:4129–44. doi: 10.1158/0008-5472.CAN-20-0471, PMID: 32816860

[72] Huang X-Y Shen J-Y Huang K Wang L Sethi G Ma Z . Cuproptosis in cancers: Function and implications from bench to bedside. BioMed Pharmacother. (2024) 176:116874. doi: 10.1016/j.biopha.2024.116874, PMID: 38850661

[73] Maji D Oh D Sharmah Gautam K Zhou M Kao J Giblin D . Copper-catalyzed covalent dimerization of near-infrared fluorescent cyanine dyes: synergistic enhancement of photoacoustic signals for molecular imaging of tumors. Anal Sens. (2022) 2:e202100045. doi: 10.1002/anse.202100045, PMID: 37621644 PMC10448761

[74] Yang N Guo X Ding J Wang F Liu T Zhu H . Copper-64 based PET-radiopharmaceuticals: ways to clinical translational. Semin Nucl Med. (2024) 54:792–800. doi: 10.1053/j.semnuclmed.2024.10.002, PMID: 39521713

[75] Acevedo KM Hayne DJ McInnes LE Noor A Duncan C Moujalled D . Effect of structural modifications to glyoxal-bis(thiosemicarbazonato)copper(II) complexes on cellular copper uptake, copper-mediated ATP7A trafficking, and P-glycoprotein mediated efflux. J Med Chem. (2018) 61:711–23. doi: 10.1021/acs.jmedchem.7b01158, PMID: 29232129

[76] Lei Y He X Li J Mo C . Drug resistance in hepatocellular carcinoma: theoretical basis and therapeutic aspects. Front Biosci (Landmark Ed). (2024) 29:52. doi: 10.31083/j.fbl2902052, PMID: 38420802

