# Supplementary material for: Targeting copper death-related long non-coding RNAs: a novel strategy to overcome immunotherapy resistance in liver cancer
Source: Front Immunol. 2026 Mar 2;17:1743964. doi: 10.3389/fimmu.2026.1743964 (PMC12989404; doi:10.3389/fimmu.2026.1743964)
Supplement: Supplementary file 1 [file Supplementaryfile1.docx]

Supplementary Material

**
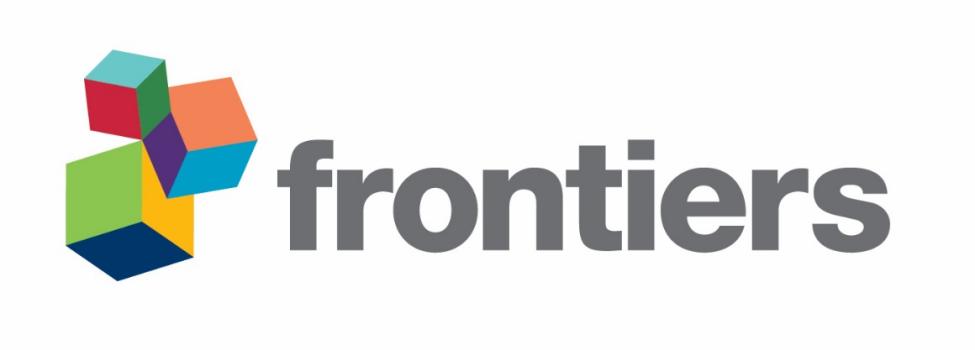
**

**Supplementary**

**Table 1: Comparison of Different Types of Cell Death**

| Types of Cell Death | Definition | Characteristics | Key Markers |
| --- | --- | --- | --- |
| Cuproptosis (25,26) | Cuproptosis is a novel form of cell death regulation triggered by intracellular copper accumulation | Mitochondrial atrophy, mitochondrial membrane rupture | FDX1,DLAT,LIAS,HSP70 |
| Ferroptosis (27,28) | Ferroptosis is a form of cell death induced by iron accumulation and oxidative stress | Mitochondrial atrophy, mitochondrial membrane rupture, reduced mitochondrial cristae | Fe,GSH,MDA,GPX4,ROS |
| Apoptosis(29) | Apoptosis is a programmed cell death process mediated by apoptotic bodies and caspases, aimed at maintaining cellular homeostasis | cell shrinkage, chromatin condensation, membrane integrity preserved, apoptotic body formation | P53,BCL-2 |
| Pyroptosis(30) | Pyroptosis is a form of programmed cell death triggered by inflammasomes | Cell swelling, cell membrane rupture, DNA condensation and fragmentation, release of pro-inflammatory cytokines | IL-18, Caspase 1, Gasdermin D, IL-1 |
| Necroptosis(31,32) | A programmed form of necrosis, mediated by RIPK1, RIPK3, and MLKL | Cell swelling, plasma membrane rupture, leakage of intracellular contents, strong inflammatory response | p-RIPK1, p-RIPK3, p-MLKL, MLKL oligomers |
| Autophagy(33,34) | A catabolic process involving lysosomal degradation of cytoplasmic components; excessive autophagy can lead to cell death | Extensive cytoplasmic vacuolization (formation of autophagosomes), organelle degradation, lack of chromatin condensation | LC3-II (conversion from LC3-I), Beclin-1, p62 (degradation) |

**Table 2: Expression profiles, prognostic associations, and potential functional mechanisms of key copper death-related lncRNAs (CRLs) in cancer.**

| LncRNA Name | Expression Status | Association of High Expression with Prognosis | Primary Functional Mechanism |
| --- | --- | --- | --- |
| MKLN1-AS(43,53,54) | Significantly Upregulated | Poor | Acts as a ceRNA for miR-654-3p, upregulating HDGF expression to drive HCC malignant progression; serves as a key component of CRDELSig; its knockdown upregulates FDX1, participating in copper-mediated apoptosis |
| AC026412.3(55) | Upregulated | Poor | Key member of CRL prognostic signature; predicts immunotherapy response |
| TMCC1-AS1(56) | Upregulated | Poor | Promotes HCC cell proliferation, migration, invasion, and EMT; common component in multiple prognostic models |
| AL133243.2(53) | Upregulated | Adverse | High-risk group characteristic lncRNA; positively correlated with immune checkpoint expression; inhibits NK cell activation |
| KDM4A-AS1(52,54) | Significantly Upregulated | Poor | Promotes tumor growth and metastasis via miR-411-5p/KPNA2/AKT axis; as a key component of CRDELSig, its knockdown upregulates FDX1 and participates in Cuproptosis processes |
| LINC02362(51) | Downregulated | Good | Sponges miR-18a-5p to upregulate FDX1; *promotes* cuproptosis and enhances oxaliplatin sensitivity. |

**Table 3**. Cross-cancer comparison of key verified CRLs: Mechanisms and functional heterogeneity.

| Cancer Type | Representative CRL | Expression Pattern | Core Molecular Mechanism | Role in Cuproptosis | Immune/Therapeutic Implication | Ref. |
| --- | --- | --- | --- | --- | --- | --- |
| HCC | LINC02362 | Downregulated | Sponges miR-18a-5p to upregulate FDX1 | Promoter (Sensitizer) | Enhances sensitivity to oxaliplatin and copper ionophores | (51) |
| HCC | MKLN1-AS | Upregulated | Sponges miR-654-3p to upregulate HDGF | Inhibitor (Resistor) | Promotes immune exclusion; drives progression | (54) |
| NSCLC | LINC01128 | Upregulated | Sponges miR-576-5p to regulate CDKN3 | Regulator | Associated with reduced CD8+ T cell infiltration | (61) |

Abbreviations: HCC, Hepatocellular Carcinoma; NSCLC, Non-Small Cell Lung Cancer; FDX1, Ferredoxin 1; HDGF, Hepatoma-Derived Growth Factor; CDKN3, Cyclin-dependent kinase inhibitor 3.

Table 4**:Representative nanodelivery systems developed for targeting cuproptosis.**

| Nano-system composition | Targeting strategy | Mechanism of action | Advantages |
| --- | --- | --- | --- |
| Polyethylene glycol-capped copper(I) oxide nanocomposite (PEG@Cu₂O-ES) (25) | EPR effect & near-infrared photothermal response | Releases Elesclomol and Cu₂O; photothermal effect enhances copper release and inhibits ATP-copper pump; induces copper-mediated cell death and sensitizes anti-PD-1 therapy | Synergistic photothermal therapy and immune reprogramming |
| Platelet-Encapsulated Cu₂O/TBP-2 System (PTC) (68) | Platelet membrane-targeted tumor delivery | Acidic-conditioned Cu⁺ release; light-induced ROS depletion of GSH, blocking copper efflux; induces potent copper-mediated death and inhibits lung metastasis | High biocompatibility with prolonged circulation and immune memory activation |
| Macrophage Membrane-Coated Cu@ZIF-8 Sonosensitizer System (SonoCu) (67) | Macrophage membrane integrin targeting | Synergistic copper-mediated cell death with sonodynamic therapy; alleviates tumor hypoxia; depletes GSH and induces mitochondrial dysfunction | Hypoxia improvement and multi-pathway cell death induction |
| Copper-doped Au@MSN nanoplatform loaded with DSF (Au@MSN-Cu/PEG/DSF) (67) | EPR effect & near-infrared light-controlled release | Photothermal triggering releases DSF and Cu²⁺, generating CuET in situ; induces Cuproptosis and apoptosis, synergizing with photothermal therapy | Precise controlled release with minimal damage to normal tissues |
| Polymer-Coated Nanoparticles Encapsulating ES and Copper (NP@ES-Cu) (67) | ROS-Responsive Release | Releases ES and Cu in high-ROS tumor microenvironments, inducing mitochondrial copper accumulation and copper-induced cell death; synergizes with αPD-L1 for enhanced immunotherapy | Suitable for immunosuppressive tumor microenvironments |

**
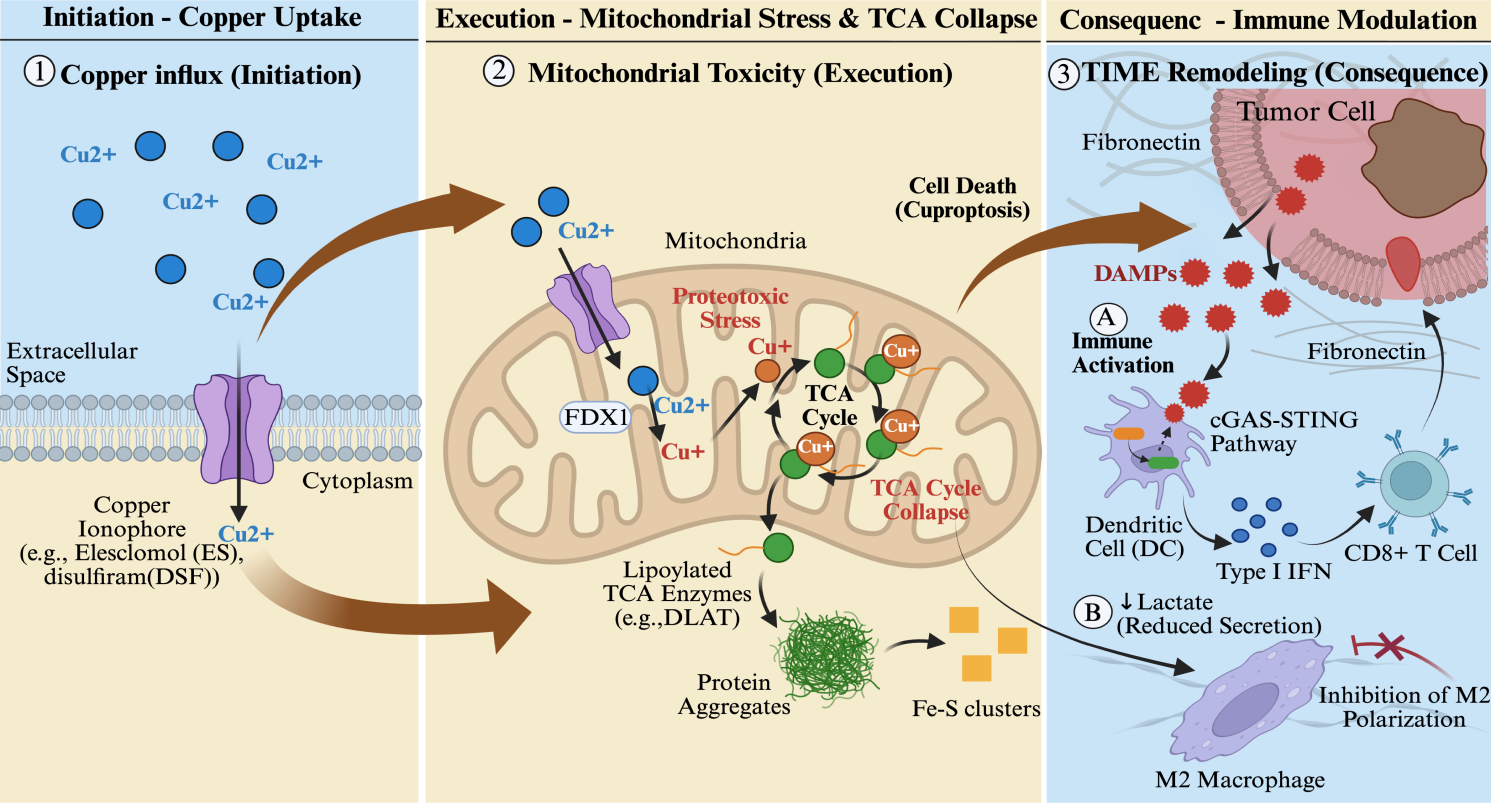
**

**Figure 1. Mechanism of copper-induced cell death and its remodeling of the tumor immune microenvironment**

The mechanism is illustrated in three sequential phases: (1) Initiation - Copper Uptake (Left): Copper ionophores, represented by purple transmembrane channels (e.g., elesclomol (ES) and disulfiram (DSF)), facilitate the influx of extracellular Cu²⁺ (blue spheres) into the cytoplasm and subsequently into the mitochondria. (2) Execution - Mitochondrial Toxicity (Center): Within the mitochondrion, FDX1 reduces Cu²⁺ to Cu⁺ (orange spheres). The highly reactive Cu⁺ selectively binds to lipoylated TCA cycle enzymes (depicted as green spheres with lipid tails, e.g., DLAT). This binding triggers the formation of insoluble protein aggregates (shown as green tangled structures) and the loss of Fe-S clusters (yellow squares), leading to proteotoxic stress and the physical collapse of the TCA cycle. (3) Consequence - Immune Modulation (Right): The rupture of the dying tumor cell releases DAMPs (red spiky shapes), which are engulfed by Dendritic Cells (DCs) to activate the cGAS-STING pathway, promoting Type I IFN secretion and CD8⁺ T cell infiltration (Path A). Concurrently, the collapse of the TCA cycle results in reduced lactate secretion (↓ Lactate), thereby attenuating lactate-driven M2 polarization / inhibiting M2 polarization (Path B).

**
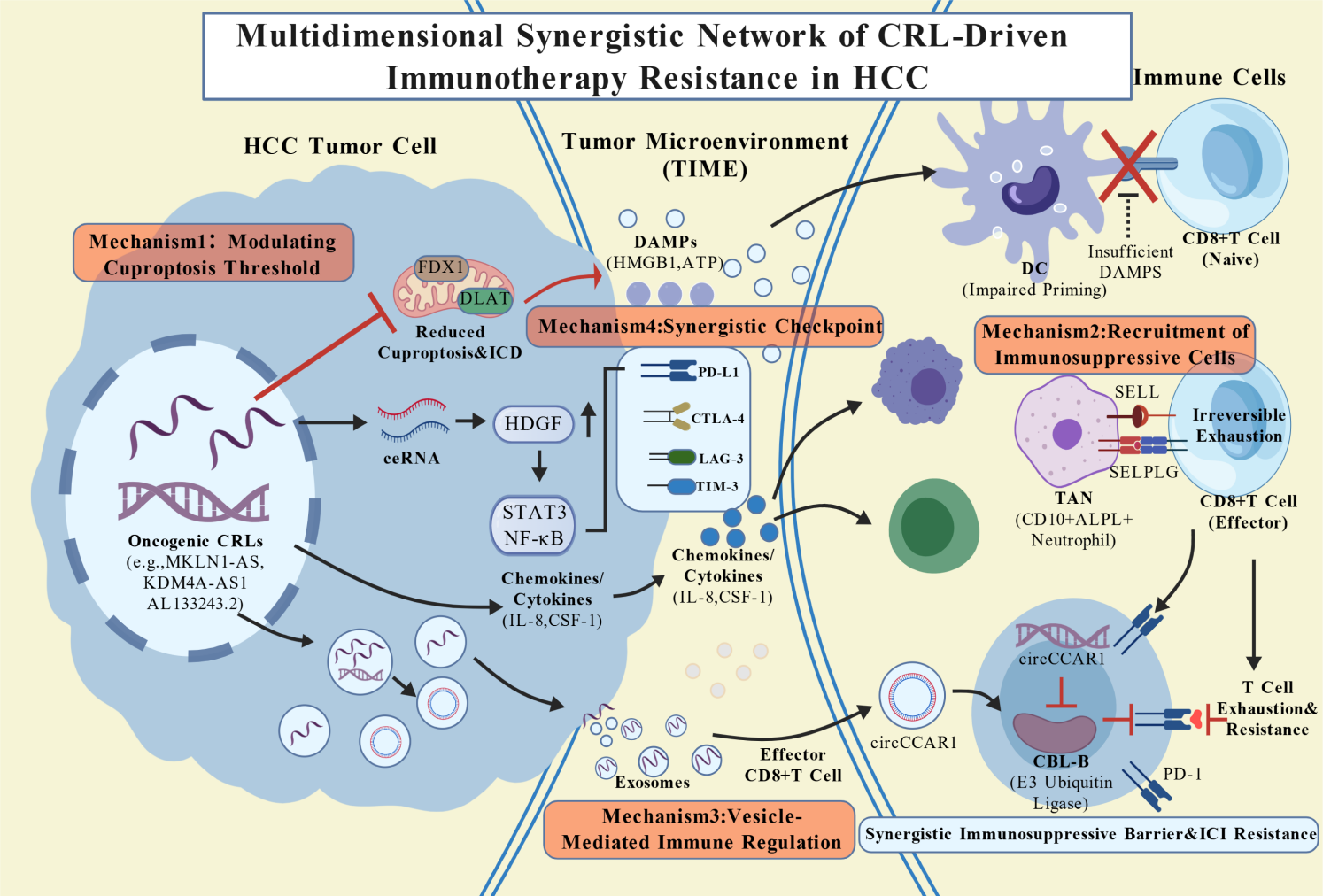
**

**Figure 2. Schematic diagram of the multidimensional mechanism by which copper death-associated lncRNAs drive immune resistance in hepatocellular carcinoma**

Mechanism 1 (Top Left/Right): CRLs downregulate cuproptosis machinery (FDX1/DLAT), reducing ICD and DAMPs release, impairing DC-mediated T cell priming.

Mechanism 2 (Middle): CRLs drive cytokine networks to recruit suppressive cells (TANs, M2, Tregs). TANs directly induce T cell exhaustion via SELL-SELPLG interaction.

Mechanism 3 (Bottom Right): Exosomal CRLs (e.g., circCCAR1) transfer to T cells, inhibiting CBL-B to stabilize PD-1 receptors, driving exhaustion.

Mechanism 4 (Integrated Surface): CRLs synergistically upregulate inhibitory checkpoints (e.g., PD-L1 on tumor, PD-1 stability on T cells), creating a multidimensional immune barrier.
